# The Bent-Tube Nozzle Optimization of Force-Spinning With the Gray Wolf Algorithm

**DOI:** 10.3389/fbioe.2021.807287

**Published:** 2021-12-15

**Authors:** Kang Liu, Wenhui Li, Peiyan Ye, Zhiming Zhang, Qiaoling Ji, Zijun Wu

**Affiliations:** ^1^ Hubei Digital Textile Equipment Key Laboratory, Wuhan Textile University, Wuhan, China; ^2^ School of Mechanical Engineering and Automation, Wuhan Textile University, Wuhan, China

**Keywords:** force-spinning, nanofibers, spinneret, optimization, gray wolf algorithm

## Abstract

Force-spinning is a popular way to fabricate various fine fibers such as polymer and metal nanofibers, which are being widely employed in medical and industrial manufacture. The spinneret is the key of the device for spinning fibers, and the physical performance and morphology of the spun nanofibers are largely determined by its structure parameters. In this article, the effect of spinneret parameters on the outlet velocity is explored and the spinneret parameters are also optimized to obtain the maximum outlet velocity. The mathematical model of the solution flow in four areas is established at first, and the relationship between outlet velocity and structure parameters is acquired. This model can directly reflect the flow velocity of the solution in each area. Then, the optimal parameters of outlet diameter, bending angle, and curvature radius are obtained combined with the gray wolf algorithm (GWA). It is found that a curved-tube nozzle with a bending angle of 9.1°, nozzle diameter of 0.6 mm, and curvature radius of 10 mm can obtain the maximum outlet velocity and better velocity distribution. Subsequently, the simulation is utilized to analyze and compare the velocity situation of different parameters. Finally, the fiber of 5 wt% PEO solution is manufactured by a straight-tube nozzle and optimized bent-tube nozzle in the laboratory, and the morphology and diameter distribution were observed using a scanning electron microscope (SEM). The results showed that the outlet velocity was dramatically improved after the bent-tube parameters were optimized by GWA, and nanofibers of better surface quality could be obtained using optimized bent-tube nozzles.

## Introduction

Nanofibers are elongated fibers ranging between 10 and 1,000 nm in diameter (Agarwal et al., 2013; Kenry and Lim et al., 2017). They have different properties compared with the materials used to make them in terms of light, thermal, magnetic, and electrical properties, and they have many excellent properties such as high porosity, excellent mechanical properties, and high surface area ratio (Li et al., 2011; Wu et al., 2016). Therefore, this method has been highly valued by researchers majoring in fiber preparation. The unique properties of nanofibers make them increasingly widely utilized in tissue engineering scaffolds, high-performance filtration media, membrane materials, plate media, electronics, biological products, and composite reinforcement materials (Barnes et al., 2007; Wang et al., 2007; Zhang et al., 2013; Hu et al., 2014; Wang et al., 2017). At present, the preparation methods of nanofibers are melt blowing, microphase separation, template synthesis, self-assembly, and electrospinning (Huang et al., 2019). Due to the disadvantages of limited optional materials, low production efficiency, and high equipment requirements of the above methods, the force-spinning method has been studied by more scholars as a novel technology (Padron et al., 2013). Compared with conventional melt spinning and electrospinning, force-spinning does not require heating and insulating the spinning material during spinning or the addition of external electric fields; therefore, its operability is also relatively simple, and it has various spinning material choices.

The nanofibers fabricated by force-spinning show excellent performance in the medical industry. The polyhydroxy butyrate valerate (PHBV) fibers prepared by fast-centrifuge spinning expressed good biocompatibility (Upson et al., 2017). Nylon-6 nanofiber membranes that inhibited bacteria growth were developed, which suggested that they could be used as wound dressings (Mandana et al., 2019a). Comparing the nanofibers with platelets prepared by electrospinning and centrifugal spinning, the porous 3D structure of centrifugal spinning fibers enabled higher cell proliferation rates (Lukášová et al., 2019). Carbon nanofibers were prepared by centrifugal spinning using polyacrylonitrile, and the microstructural and electrochemical properties of the prepared samples were studied (Zhao et al., 2017).

The following studies investigated battery electrodes because of the good electrical performance of the nanofibers. Antimony tin alloy (SnSb) was considered a promising sodium-ion battery negative electrode material owing to its large capacity (Hao et al., 2018). Nanofibers made from one material are called single fibers and those made from multiple materials are called composite fibers, which show more excellent performance. Composite fibers prepared with polyethylene pyrrolyl ketone (PVP) and polyethylene glycol (PEG) had excellent thermal properties (Zhang et al., 2018). SnSb@carbon microfiber complexes as a high-performance anode of sib could maintain their structural stability in repeated charge and discharge cycles (Hao et al., 2019). PS/SiO2 composite nanofiber had thermal insulation properties, and it could be a new type of building thermal insulation material (Leng et al., 2019).

At the same time, the metal fibers were also prepared by force-spinning. Hollow hematite (α-Fe_2_O_3_) fine fibers with multiwall structures were synthesized by fast-centrifugal spinning (Mandana et al., 2019b). SnSb alloy porous carbon fiber (PCF) composite was prepared with a small particle size and uniform distribution (Ning and Li, 2021).

Some studies have focused on the effect of spinning parameters on fibers. The effect of various spinning parameters on the synthesis of alumina fibers with a diameter of 5–15 microns was studied, and the effect of rotation speed and viscosity on the quality of spun fibers was examined (Thamarai and Parag, 2018). The diameter distribution state of nanofibers was researched by changing the nozzle diameter (Lu et al., 2013). The effect of nozzle direction on the initial jet motion (Zhmayev et al., 2015) and the effect of nozzle length on jet stability through the simulation of the solution motion of the force-spinning nozzle were explored (Chen et al., 2020). According to the previous studies, several researchers have begun to seek the parameters most suitable for centrifugal spinning for fiber fabrication. Four different spinning nozzles were proposed and the curved-tube nozzle was found to be more suitable for high-speed centrifugal spinning (Lai et al., 2021). Based on the complex network dynamics model in the context of derived topics, the multidimensional public opinion process was modeled (Chen et al., 2021). Therefore, this article further optimizes the curved-tube nozzle parameters that affect the surface quality of the fiber morphology based on the previous research. First, the mathematical model of the solution was established by analyzing the flow state of the solution, and second, the structural parameters (bending angle, outlet diameter, and curvature radius) were optimized combined with the GWA to obtain the optimal parameters. Then, the correctness of the optimization results was tested by simulation. Finally, the nanofibers were spun in the laboratory and the fiber morphology and surface quality were observed by SEM. The results show that the optimized curved-tube nozzle can spin fibers of excellent morphology with better surface quality and smaller diameter distribution.

## The Literature of the Gray Wolf Algorithm

In order to further explore the influence of spinning equipment on fiber morphology and quality, an optimization algorithm is used to design the spinning nozzle parameters. The gray wolf algorithm simulates the predation behavior of gray wolves and can optimize the global search through the wide distribution of wolves; thus, it is utilized to optimize the spinning solution flow model to obtain the influence of equipment parameters at a certain concentration.

Yang et al. (2007) presented the Wolf Group Search (WGS) based on the survival of wolf populations in nature and utilized it into the local search process of Marriage in Honey Bees Optimization algorithm. Oftadeh et al. (2010) proposed a hunting research (HuS) algorithm inspired by group hunting of animals. Mirjalili et al. (2014) put forward a novel population intelligent optimization method, the gray wolf optimizer (GWO), which imitated the leadership hierarchy and hunting activity of gray wolves in nature.

**FIGURE 1:**
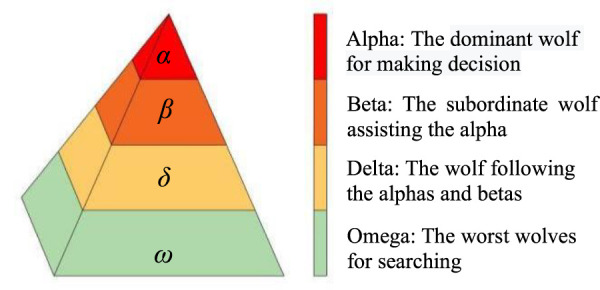
Leadership hierarchy and characteristics of wolves.

A gray wolf family consists of several or a dozen wolves, and there is an obvious hierarchy in the family members. The gray wolves are usually divided into four types: alpha, beta, delta, and omega, simulating their leadership hierarchy, as shown in Figure 1.

The top floor of the pyramid is *α* wolf, the most significant individual in the wolf family, and its work is mainly responsible for decision-making. The second floor of the pyramid is *β* wolf, the military counselor of the whole team, and its duty is to assist *α* in making management decisions. The third floor of the pyramid is known as the *δ* wolf and it follows *α* and *β* wolf instructions, but it can also command the inferior wolves. The bottom level of the pyramid is called the *ω* wolf, which accounts for the most number of wolves, whose duty is undertaking the main search tasks, and the key to the algorithm is how to utilize the *ω* wolf search capability fully. The algorithm was applied and improved in different domains in subsequent studies.

The GWO is quickly applied to various optimization problems due to the ease of operation and the ability to quickly seek optimal values. A modified Recurrent Neural Network with an adapted GWO was used to forecast students’ outcomes and functioned as an early warning system (Tarik et al., 2019). The GWO was used to optimize the super defect photonic crystal (PhC) filter, and the advantages of this method in simplifying the design process of PhC filter and seeking high-performance design are proved (Chaman-Motlagh, 2015). The gray wolf optimizer combined with a pattern search algorithm solved the problem of the management of smart grid power system security under critical conditions (Mahdad and Srairi, 2015). The algorithm was utilized to solve the non-convex dynamic economic load scheduling problem of the electric power system, and the results showed that the proposed algorithm could provide a very competitive search ability (Kamboj et al., 2016). A real-world scheduling problem in welding production was solved through an effective multi-objective discrete gray wolf optimizer (Lu et al., 2016). The maximum power point tracking (MPPT) design of the photovoltaic system adapted the gray wolf optimization technology (Mohanty et al., 2016). The GWO method was better suitable for UAV two-dimensional path planning problems than other optimization methods (Zhang et al., 2016).

Many researchers also paid attention to the application of GWO in practical problems combined with other algorithms and improving the traditional GWO. A systematic and meta-analysis survey of whale optimization algorithm (WOA) was conducted, and the statistical results of WOA modifications and hybridizations were established (Hardi et al., 2019). Then, a hybrid of WOA and GWO called WOAGWO was proposed, and it was found that WOAGWO achieved the optimum solution that was superior to WOA and GWO (Hardi and Tarik, 2020). The evolutionary population dynamics (EPD) removed the GWO search agents and relocated them around alpha, beta, or delta wolves to enhance development (Saremi et al., 2015). The hybrid PSO-GWO algorithm based on swarm intelligence had better performance than the particle swarm optimization algorithm in solving single region unit input problems (Saremi, 2016). The K-GWO, combining GWO with a traditional K-means clustering algorithm, solved the capacitated vehicle routing problem (Korayem et al., 2015). A new algorithm using K-means clustering to improve GWO performance was called K-means clustering Gray algorithm Wolf Optimization (KMGWO) (Hardi et al., 2021). A mechanism based on a mutation operator and an eliminating-reconstructing mechanism for wolves with poor search not only expanded the random search but also increased the convergence rate (Zhang and Ming, 2018). In order to eliminate the waiting time, two dynamic GWO algorithms were proposed, in which the position vector of the former search wolf could be updated after the comparison between itself or the previous searcher wolf and the leader (Zhang et al., 2021).

Considering that this algorithm is applied to the optimization of spinning equipment for the first time, the traditional gray wolf algorithm was adopted to optimize the model.

## The Flow Model of Spinning Solution in Spinneret

**FIGURE 2:**
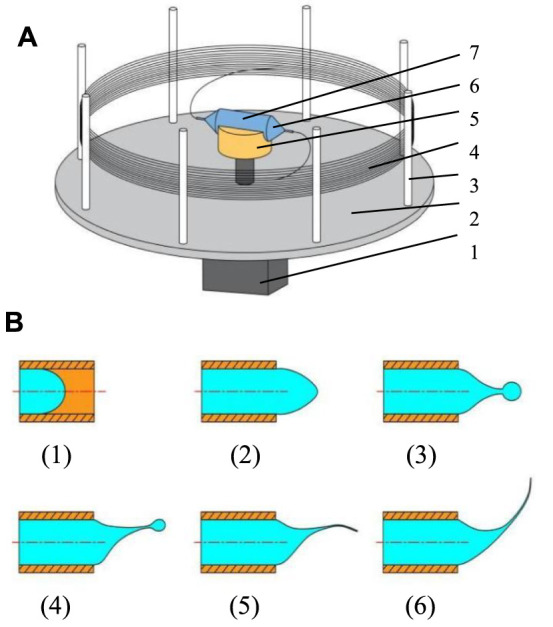
Diagrammatic sketch of the spinning device and solution ejection. **(A)** Structure of the spinning device. **(B)** Ejection process of spinning solution. 1, electric motor; 2, collecting plate; 3, collecting column; 4, nanofibers; 5, connecting part; 6, nozzle; 7, container.

The structure of the force-spinning device is shown in Figure 2A. The equipment mainly consists of two nozzles, a container, several collecting columns, a collecting plate, and an electric motor. The nozzles and container form the spinneret. The spinning solution in the container rotates with the electric motor rotation and then moves toward the nozzle under the inertial force generated by motor rotation. The process of the solution ejection and the fiber formation is shown in Figure 2B. As the speed of the motor increases, the solution gradually forms small droplets in the orifice of the nozzle head (Figure 2B (3)).

The spinning solution droplets will be ejected from the orifice and then move in the air to form the jet when the centrifugal force is large enough to conquer the viscous force and surface force of the solution. Subsequently, the jet stretches and becomes fine to form fibers with the solvent evaporating. Finally, the fibers are collected by collecting columns mounted on the collecting plate. The force-spinning spinneret is the vital core of this technique, and its structure parameters are directly related to the quality, property, and morphology of fabricated nanofibers.

**FIGURE 3:**
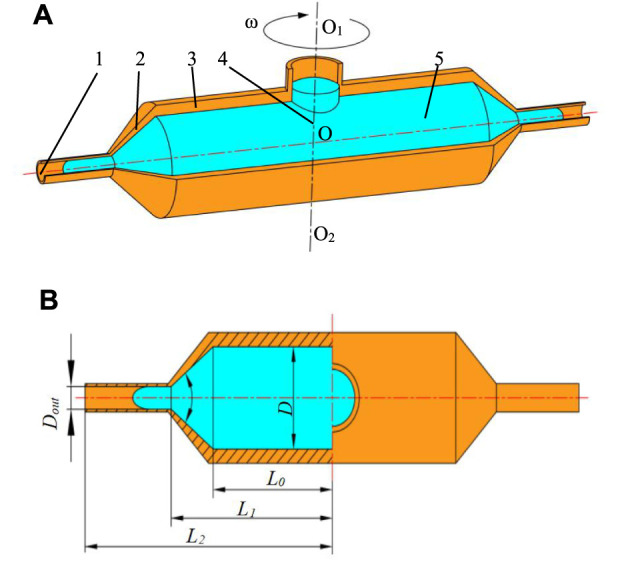
Structure schematic diagram and dimensions of the spinneret. **(A)** Structure schematic diagram of the spinneret. **(B)** Structural dimensions of the spinneret. 1, nozzle outlet; 2, nozzle; 3, container; 4, rotation axis; 5, spinning solution.

The mathematical model of the solution outlet velocity was obtained by analyzing the flow of the spinning solution in the container, nozzle, straight tube, and bent tube. The structure schematic diagram of the spinneret is shown in Figure 3A. The spinneret consists of a container and two nozzles. The container rotates around the O_1_O_2_ axis and the solution and nozzles rotate with the container. The structural dimensions of the components are shown in Figure 3B. The internal diameter of the container is D, the distance from the section of the nozzle contraction to the axis of rotation is L_0_, the distance between nozzle straight-tube inlet to the rotation shaft is L_1_, and the distance from nozzle outlet to the rotation axis is L_2_, and the contractile angle is *α*.

**FIGURE 4:**
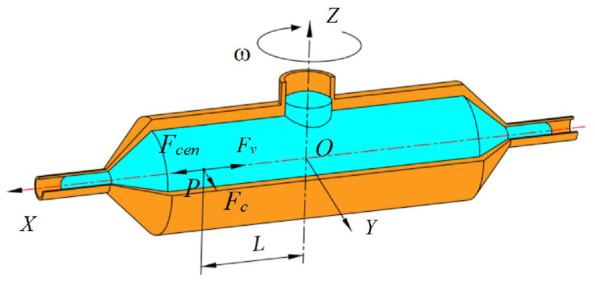
Cartesian coordinate system of the spinneret.

The Cartesian coordinate system is established at the intersection point *O* of the container center axis and the rotation axis, where the container axis is the *X*-axis and rotation axis is *Z*-axis, and the Cartesian coordinate system is shown in Figure 4. The solution at point *P*, with the distance *L* from the rotation axis, is subjected to the centrifugal force *F*_*cen*_, the viscosity force *F*_*v*_ and the Coriolis force *F*_*c*_ in the non-inertial coordinate system when the spinning device works. The direction of centrifugal force points to the positive direction of the *X*-axis, the viscous force to the negative direction of *X*-axis, and the Coriolis force to the opposite direction of rotation.

**FIGURE 5:**
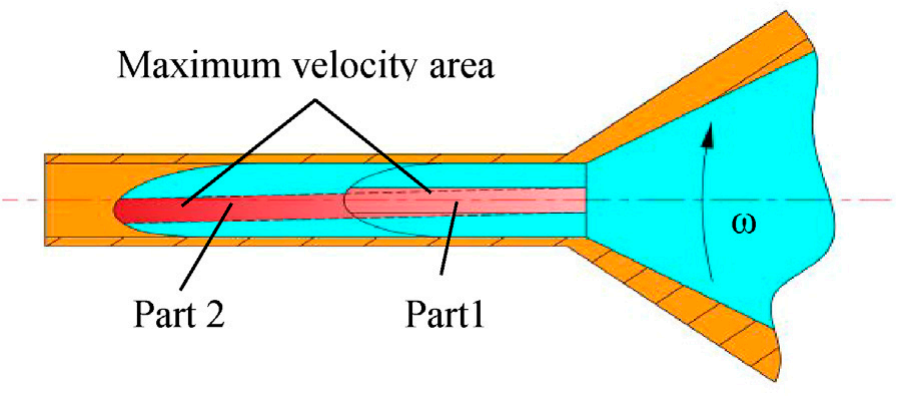
Solution flow change in the spinneret.

The solution flow in the spinneret will change compared with the nonworking state flow because of the Coriolis force existing. Figure 5 shows the flow change of the solution in the spinneret, where part 1 is the maximum solution flow velocity area under nonworking state flow and the maximum flow velocity area in working will transform into part 2. It is obvious that the maximum solution flow velocity area has a motion trend in the opposite direction of the rotational velocity; thus, when the solution is ejected from the spinneret outlet, the solution flow rate on one side of the symmetric axis is faster than the other side, which leads to the uneven distribution of the formed fiber mass and even the uneven fiber stress distribution in the production of metal fibers. The surface quality of nanofibers is also influenced owing to the uneven flow velocity. The modification of the spinneret structure can concentrate the solution's maximum velocity area at the outlet center (Lai et al., 2021).

**FIGURE 6:**
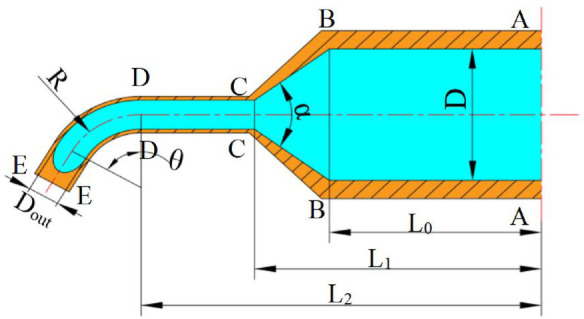
Solution flow state in four areas.

The experiment found that adding a bent tube at the outlet of the nozzle straight pipe, making the solution velocity center change at the bend, could significantly improve the velocity distribution. Figure 6 shows the solution flow state at four areas in the additional bent tube. Section A-A to section B-B is the container area, section B-B to section C-C is the nozzle contraction section, section C-C to section D-D is the straight tube area, and section D-D to section E-E is the bent tube area.

The solution micro-mass is *m* and the velocity along with the centrifugal force *F*_*cen*_, viscous force *F*_*v*_, and Coriolis force *F*_*c*_ container axis is *V* at point *P*; therefore, the equations of *F*_*cen*_, *F*_*v*_, and *F*_*c*_ are, respectively, represented as follows:

$$\{\begin{array}{c} F_{cen}=m\omega^{2}L\\ F_{v}=k{\left(\frac{\partial V}{\partial r}\right)}^{n}\\ \vec{F}c=2m\vec{V}\times \vec{\omega } \end{array},$$ (1)

where ∂V/∂r is the velocity gradient of the spinning solution, *k* is the viscosity coefficient of the spinning solution, and *n* is the rheological index of the spinning solution, representing the rheological characteristics of the spinning solution. *k* and *n* both are related to the solution concentration, which can be obtained through the data measured by rheological experiments. $\vec{V}$ is the relative velocity of the solution at p point along the *X*-axis. $\vec{\omega }$ is the vector of the motor rotation angular velocity *ω*. The size of Coriolis force is $|\vec{F_{c}}|=2m\omega v\text{sin}90{}^{\circ}$ .

The solution velocity along the axis of the container is very low when the spinning device starts to work, so the viscous force is very small. The solution in the container is mainly subjected to the centrifugal force and Coriolis force. The Coriolis forces are balanced with the intermolecular forces and the reaction forces of the container wall. When the spinning device works stably, the cross section is fully filled with solution; therefore, the effect of the Coriolis force on the container axis is negligible. The solution flows toward the nozzle under the centrifugal force. The solution flow in the container area can be seen as a steady flow because when the device works steadily, the velocity, pressure, temperature, and density at any point in the flow channel are constant.

In the non-inertial coordinate system, the solution flow in the container is in parallel linear motion. The velocity of the point *p* in the container is *V*_*p*_. According to the energy equation, it can be written as

$$\int_{0}^{L}F_{cen}-F_{v}dx=\frac{1}{2}mV_{P}^{2}.$$ (2)

The 5wt% PEO solution was used for the experiment and the value of viscosity coefficient *k* and rheological index *n* are, respectively, 7.62 and 0.502. Substituting Eq. 1 into Eq. 2, the relationship between the velocity *V*_*P*_ and the distance *L* can be known; thus, Eq. 2 can be simplified as

$$V_{p}=\frac{\omega }{10^{4}}L.$$ (3)

It is obvious that when the solution flows to section B-B, the velocity of *V*_*B*_ is obtained as

$$V_{B}=\frac{\omega }{10^{4}}L_{0}.$$ (4)

When the solution flows into the nozzle, the velocity of the solution increases with not only the effect of centrifugal force but the shrinkage of the interior diameter of the nozzle. It is not feasible to use the energy equations or the continuous equations alone. Therefore, the velocity of section *C-C* is defined as follows:

$$V_{C}=\frac{DV_{B}}{10D_{out}}.$$ (5)

As the velocity of the spinning solution increases rapidly, the effects of the viscous force and the Coriolis force also are enhanced dramatically. However, it just influenced the velocity distribution. The energy equation of solution in straight tube along the *X*-axis is written as

$$\int_{L_{1}}^{L}F_{\text{cen}}-F_{v}dx=\frac{1}{2}m\left(V^{2}-V_{c}^{2}\right).$$ (6)

Substituting Eq. 6 into Eq. 7, the velocity of the spinning solution in the straight tube can be represented as

$$V^{2}=\frac{\omega^{2}}{10^{8}}\left(L^{2}-L_{1}^{2}\right)+\frac{D^{2}V_{B}^{2}}{10^{2}D_{out}^{2}}.$$ (7)

Hence, the velocity of solution in the straight tube is also a function of the *L*. The maximum is reached at section D-D of the straight tube. The velocity before solution flowing into bent tube is expressed as

$$V_{D}^{2}=\frac{\omega^{2}}{10^{8}}\left(L_{2}^{2}-L_{1}^{2}\right)+\frac{D^{2}V_{B}^{2}}{10^{2}D_{out}^{2}}.$$ (8)

**FIGURE 7:**
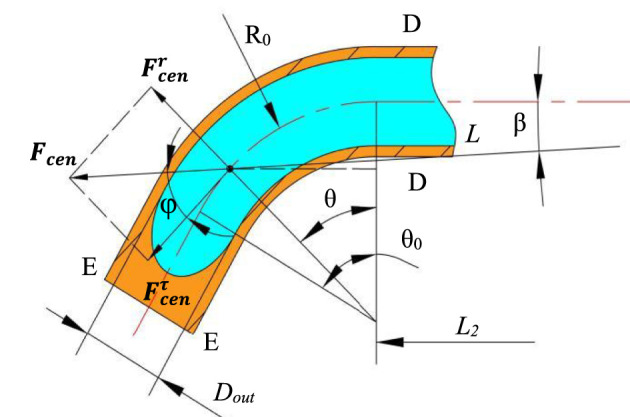
Solution flow in the bent tube.

When the solution flows into the bent tube, the direction of centrifugal force will change lightly and the solution flow in the bent tube is shown in Figure 7. The distance between the point P and rotation axis is *L*, so the centrifugal force is constructed as

$$F_{cen}=m\omega^{2}L=m\omega^{2}\sqrt{{\left(L_{2}+R\sin\varphi \right)}^{2}+{\left(R-R\cos\varphi \right)}^{2}}.$$ (9)

According to the energy equation, the velocity in the bent tube outlet is written as

$$\int_{0}^{\theta_{0}R_{0}}F_{cen}^{\tau }-F_{v}ds=\int_{0}^{\theta_{0}R_{0}}F_{cen}\cos\varphi -F_{v}ds=\frac{1}{2}m\left(V_{E}^{2}-V_{D}^{2}\right).$$ (10)

It can also be written as follows:

$$\frac{1}{2}m\left(V_{E}^{2}-V_{D}^{2}\right)=\int_{0}^{\theta_{0}R_{0}}m\omega^{2}\sqrt{{\left(L_{2}+R\sin\theta \right)}^{2}+{\left(R-R\cos\theta \right)}^{2}}\cos\varphi -k{\left(\frac{\partial V}{\partial r}\right)}^{n}ds.$$ (11)

The relationship between the angles β, *θ*, and *φ* can be expressed as

φ=θ−β. (12)

Since the angle *ß* is relatively small, it is set to 0 for computational convenience, which makes the direction of the centrifugal force point to the positive of the *X*-axis. Eq. 11 will be transformed into

$$\frac{1}{2}m\left(V_{E}^{2}-V_{D}^{2}\right)=\int_{0}^{\theta_{0}R_{0}}m\omega^{2}\left(L_{2}+R_{0}\sin\theta \right)\cos\theta -k{\left(\frac{\partial V}{\partial r}\right)}^{n}ds.$$ (13)

Substituting Eq. 8 into Eq. 13, the outlet velocity of the solution at section E-E is obtained as follows:

$$V_{E}^{2}=\frac{\omega^{2}}{10^{8}}\left[R_{0}^{2}\cos^{2}\left(\theta_{0}\right)+L_{2}R_{0}\sin\left(\theta_{0}\right)\right]+\frac{\omega^{2}L_{0}^{2}D^{2}}{10^{10}D_{out}^{2}}+\frac{\omega^{2}}{10^{8}}\left(L_{2}^{2}-L_{1}^{2}\right).$$ (14)

## Optimization for Spinneret With Curved Tube

### The Mechanism of Gray Wolf Optimizer and Its Application in Curved-Tube Spinneret

The GWO algorithm consists of three main steps: searching for prey, encircling prey, and attacking prey. When the gray wolf determines the location of the prey, the head wolf *a* will lead other wolves to chase. However, in solving the function optimization problem, the position of the prey corresponds to the global optimal solution of the problem, which is not known in advance. *α*, *β*, and *δ* are the three wolves closest to their prey; thus, the position of the *α*, *β*, and *δ* wolves can be taken as an approximate solution, where the *α* wolf is the optimal solution.

**FIGURE 8:**
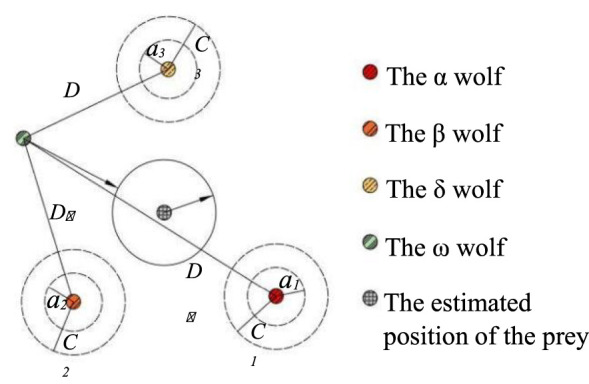
Position updating in GWO.

The mechanism by which individuals within the group track their prey orientation is shown in Figure 8 and the specific calculation can be represented as follows (Mirjalili et al., 2014):

$$\{\begin{array}{c} D_{\alpha }=\left|C_{1}\cdot X_{\alpha }-X_{\omega }^{t}\right|,\\ D_{\beta }=\left|C_{2}\cdot X_{\beta }-X_{\omega }^{t}\right|,\\ D_{\delta }=\left|C_{3}\cdot X_{\delta }-X_{\omega }^{t}\right|, \end{array}$$ (15)

$$\{\begin{array}{c} X^{t+1}=X_{\alpha }-A_{1}\cdot D_{\alpha },\\ X^{t+1}=X_{\beta }-A_{2}\cdot D_{\beta },\\ X^{t+1}=X_{\delta }-A_{3}\cdot D_{\delta }, \end{array}$$ (16)

$$X_{\omega }^{t+1}=\frac{X_{\alpha }^{t+1}+X_{\beta }^{t+1}+X_{\delta }^{t+1}}{3},$$ (17)

where *t* denotes the current number of iterations, $X_{\omega }^{t}$ is the present position vector of the *ω* wolf after *t*th iteration, and *X*_α_, *X*_*β*_, and *X*_*δ*_ are the position vectors of the *α*, *β*, and *δ* wolves, respectively. *D*_α_, *D*_*β*_, and *D*_*δ*_ are the distance between the *ω* wolf and the *α*, *β*, or *δ* wolf. *A* and *C* are the coefficient matrices, which are given as follows:

$$\{\begin{array}{c} A=2a\cdot r_{1}-a,\\ C=2r_{2},\\ a=2\left(1-\frac{t}{\text{Iteration}}\right) \end{array},$$ (18)

where Iteration is the maximum number of iterations, r_1_ and r_2_ are two random vectors in [0,1], and the value *a* decreases linearly from 2 to 0.

The flow of the algorithm is as follows: 1) the number of wolves, the problem dimension, and convergence condition are set, and the initial position of each wolf and the values of a, A, and C are randomly generated in the search space; 2) calculate the fitness value of each wolf and select the position of the *α*, *β*, and *δ* wolves according to the optimal fitness value; 3) the distances between the remaining wolves and the *α*, *β*, and *δ* wolves are obtained according to Eq. 15; 4) update the individual positions of wolves according to Eqs. 16, and 17; 5) recalculate the values of a, A, C, and fitness; 6) if the convergence is achieved, end the iteration and output the results; if not, return to step (3).

The optimization problem is solved by establishing a relationship between the parameters affecting the outlet velocity and the GWO; therefore, the gray wolf position vector consists of three pending parameters in the outlet velocity. The vector X can be defined as follows:

$$X={\left(\begin{array}{c} \text{Curvature}\;\text{radius}\\ \text{Bending}\;\text{angle}\\ \text{Nozzle}\;\text{diameter} \end{array}\begin{array}{c} R_{0}\\ \theta_{0}\\ D_{\mathrm{out}} \end{array}\right)}^{T}.$$ (19)

### Establishment of the Optimization Model

It can be obtained from Eq. 14 that the outlet velocity *V*_*E*_ can be represented as follows:

$$\frac{10^{8}}{\omega^{2}}V_{E}^{2}=R_{0}^{2}\cos^{2}\left(\theta_{0}\right)+L_{2}R_{0}\sin\left(\theta_{0}\right)+\frac{L_{0}^{2}D^{2}}{10^{2}D_{out}^{2}}+\left(L_{2}^{2}-L_{1}^{2}\right).$$ (20)

Moreover, the optimization objective function is established as follows:

$$\max F\left(x\right)=\max V_{E}.$$ (21)

The simplified fitness function can be written as follows:

$$f_{x}=R_{0}^{2}\cos^{2}\left(\theta_{0}\right)+L_{2}R_{0}\sin\left(\theta_{0}\right)+\frac{L_{0}^{2}D^{2}}{10^{2}D_{out}^{2}}.$$ (22)

**FIGURE 9:**
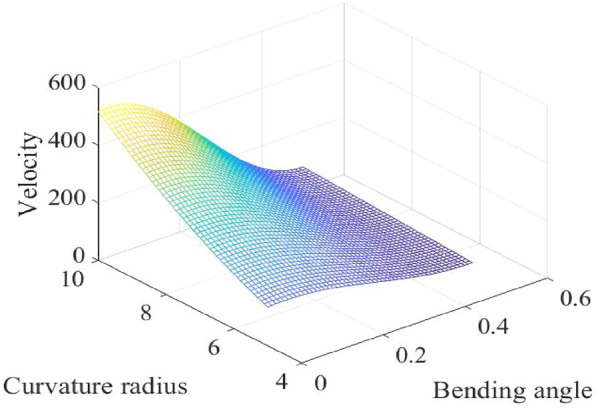
Diagram of the fitness function.

It could be found from Figure 9 that the maximum value of the outlet velocity is at the position of the larger value of curvature radius, smaller value of nozzle diameter, and bending angle.

### The Optimization Structure Parameters of Curved-Tube Nozzle

Optimization is efficiently facilitated by determining the range of the parameters. The range of optimized parameters is shown in Table 1.

**TABLE 1:** Range of optimized parameters.

Items	Parameters	Range
Optimization object	*V*_*out*_	Max
Design parameters	*R*_*0*_	[6, 10]
	*θ*_*0*_	[0,0.5] (*π*)
	*D*_*out*_	[0.6, 1.0]
Other spinneret parameters	D	20
	L_0_	30
	L_1_	45
	L_2_	55
Other parameters	Ω	3,000
	K	7.62
	N	0.502
Parameters about the GWO	Total number of wolves	20
	Maximum iteration number	50
	A	[−a, a] a = 2→0
	C	(0,2]
	Number of experiments	10

**FIGURE 10:**
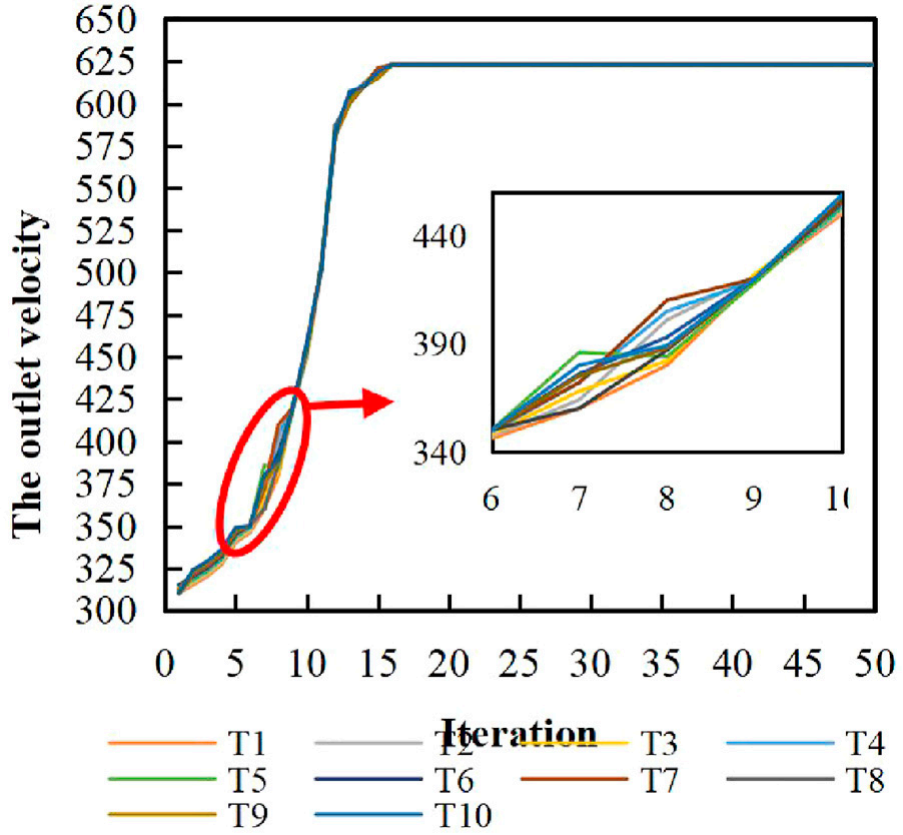
Process of GWO for optimization.

After multiple parameter adjustment and iterative operations, the GWA optimization process is shown in Figure 10. T_i_ (*i* = 1,2,…,10.) represents the process diagram of the *i*th GWO optimization. It is obvious that the optimal search converges when the number of iterations reaches 18, which shows that the GWO can effectively solve the nozzle optimization problem. It is also known that the gray wolf algorithm was in the global search stage in the early stage, and the convergence rate was slow. The optimization search speed increased rapidly while more wolf members approached the *a*, *β*, and *δ* wolves.

In ten experiments, it can be found that when the number of iterations is 6–9, some experiments can find the optimal result quickly, and some are poor. This suggests that there may be possible progress toward local optimality during the global search of wolf populations.

### The Optimization Results

The optimum nozzle structure parameter values are shown in Table 2.

**TABLE 2:** Optimum nozzle structure parameter values.

Items	Curvature	Bending angle	Nozzle diameter
Parameters	*R*_*0*_ (mm)	*θ*_*0*_ (°)	*D*_*out*_ (mm)
Value	10	9.1	0.6

## The Flow Field Simulation Experiment for Force-Spinning

### The Simulation Model of Spinneret

**FIGURE 11:**
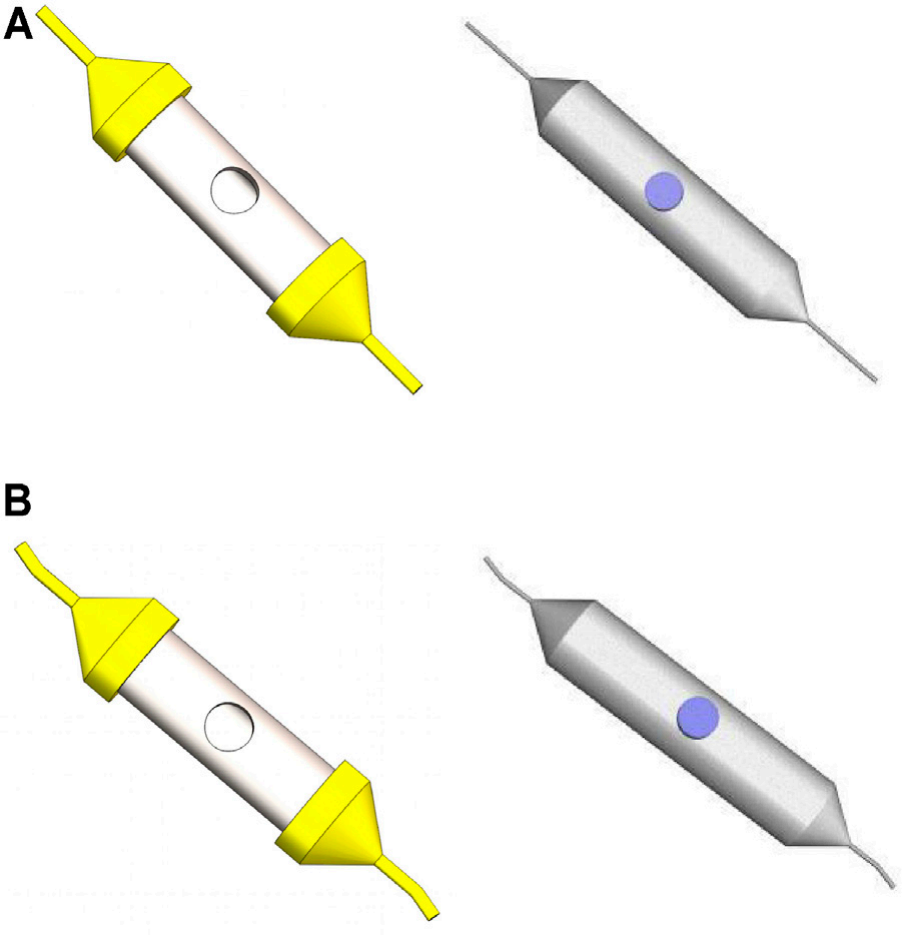
3D structure and mesh model of the straight-tube and curved-tube spinnerets. **(A)** 3D structure and mesh model of the straight-tube spinneret. **(B)** 3D structure and mesh model of the curved-tube spinneret.

The 3D structure and mesh model of the straight-tube and curved-tube spinnerets are shown in Figure 11. An unstructured grid was adopted to divide the solution flow model, and the grid structure was a tetrahedral mesh. The maximum mesh size of the straight tube and curved tube was set to 0.1 mm, and the maximum mesh size of the container and nozzles was 0.5 mm. The boundary layer was divided into three layers, and the boundary layer thickness was one-tenth of the maximum grid size of each part.

The boundary conditions of the force-spinning motion model mainly include four aspects. The inlet boundary is velocity inlet and the hydraulic diameter is set to 12 mm. The outlet boundary is the pressure outlet, and the hydraulic diameter is set to the corresponding diameter of the different nozzles. The dynamic mesh is set as the rotating reference system and the rotating axis is *Z*-axis. The rotating angular velocity is set to 3000 rpm.

### The Flow Field Motion Simulation of Spinnerets

**FIGURE 12:**
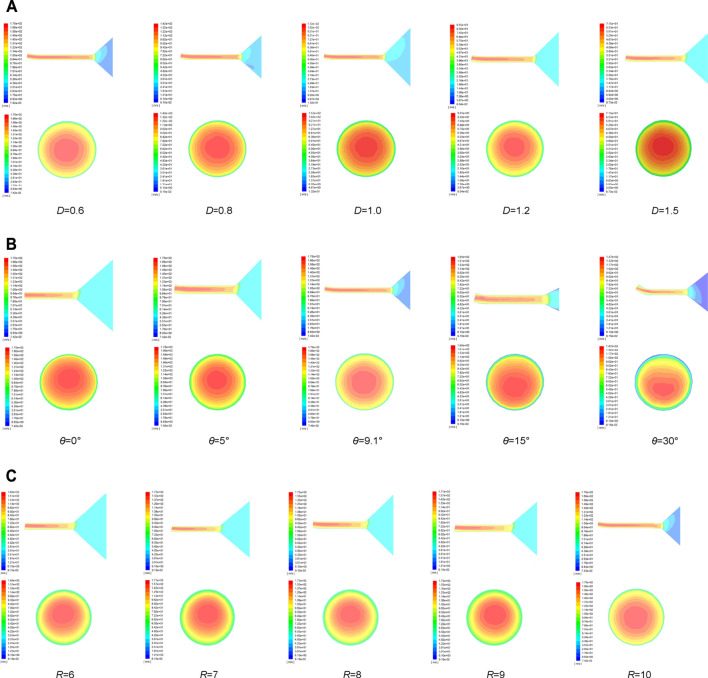
Velocity cloud map along the tube axis and velocity distribution cloud map at outlet section **(A)** *θ* = 9.1°, R_0_ = 10 mm. **(B)** *D* = 0.6 mm, R_0_ = 10 mm. **(C)** *D* = 0.6 mm, *θ* = 9.1°.

The flow field models at different bending angles, curvature radius, and outlet diameters were analyzed. The velocity gradient along the axis and the velocity distribution on the outlet cross section were used as the criterion to verify whether the best parameters optimized by the gray wolf algorithm were correct. Figure 12 shows the velocity cloud map along the tube axis and the velocity distribution at a bending angle of 9.1° and curvature radius of 10 mm, at outlet diameter of 0.6 mm and curvature radius of 10 mm, and at a bending angle of 9.1° and outlet diameter of 0.6 mm, respectively.

It can be easily known that the outlet velocity increases as the outlet diameter decreases from the velocity cloud map at the outlet section. The smaller outlet diameter can effectually improve the production efficiency. The change in the bending angle effectively concentrates the solution maximum outlet velocity on the tube axis, which made the solution distribution more even to fabricate nanofibers of high quality. The transformation of the curvature radius has little effect on the outlet velocity distribution.

**FIGURE 13:**
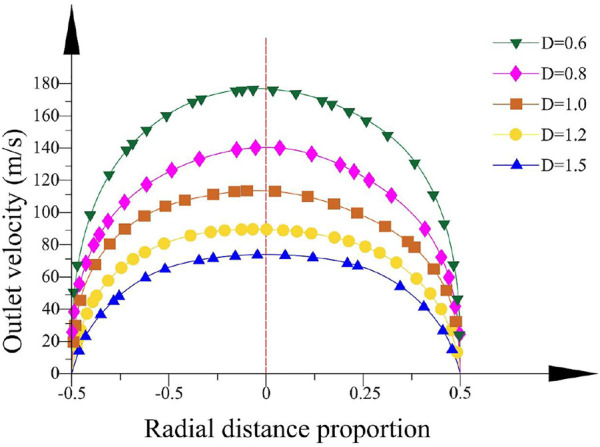
Velocity distribution in different outlet diameters.

**FIGURE 14:**
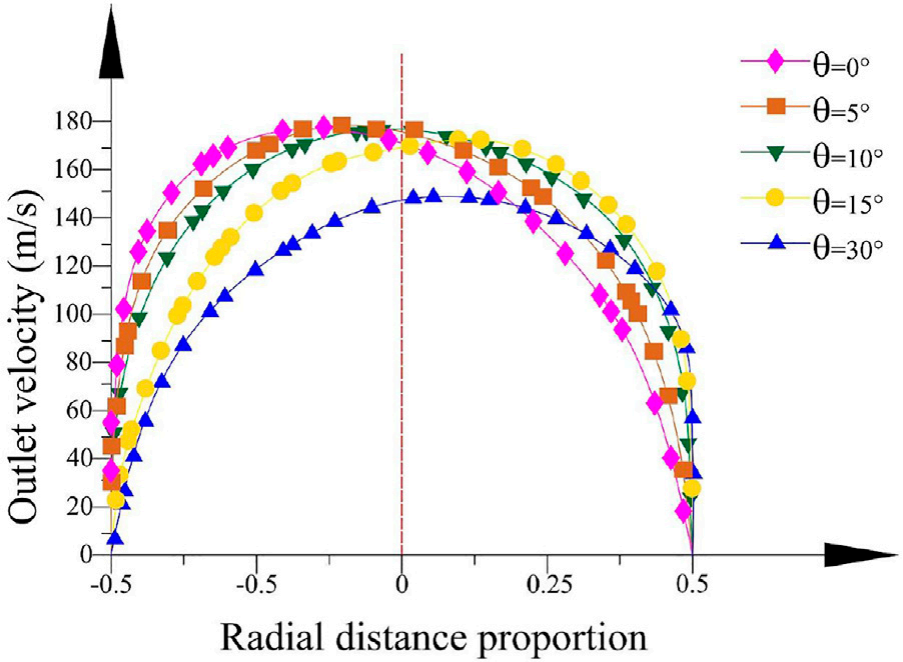
Velocity distribution in different bending angles.

**FIGURE 15:**
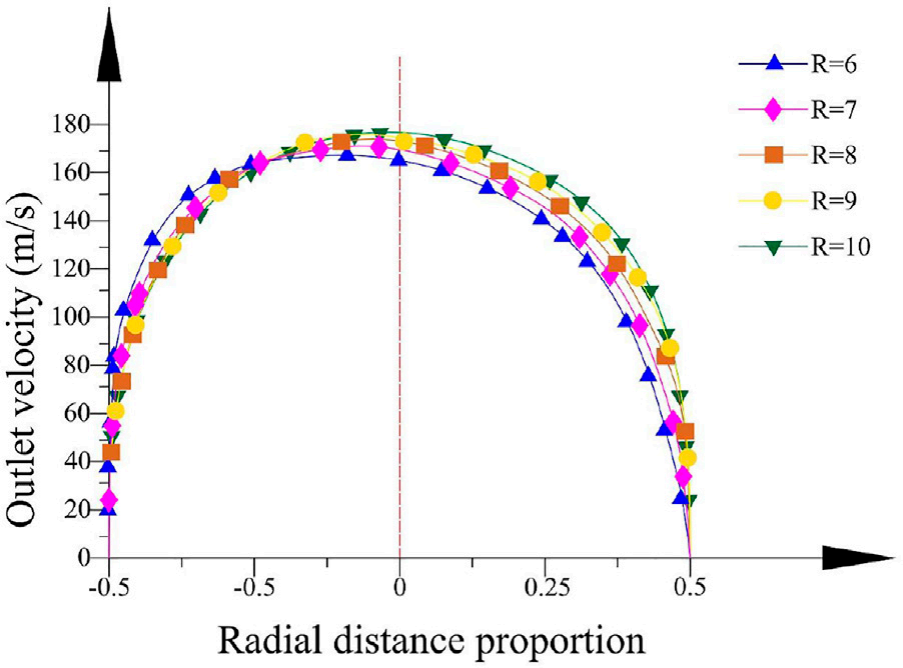
Velocity distribution in different curvature radiuses.

The velocity tracing the intersection line between the rotational horizontal plane of the container axis and the outlet section is analyzed. Figure 13 depicts the velocity distribution clearly when the bending angle *θ* is 9.1° and the curvature radius *R*_*0*_ is 10 mm. It is obviously found that the maximum outlet velocity concentrates near the axis of the bent tube and the velocity increases with the outlet diameter decreasing. Figure 14 shows the velocity distribution in outlet diameter of 0.6 mm and curvature radius of 10 mm. The changes in bending angle mainly influence the velocity distribution. Figure 15 indicates the velocity distribution in the outlet diameter D = 0.6 mm and the bending angle *θ* = 9.1°. The variations in bending angle have little effect on velocity and its distribution.

## The Force-Spinning Experiment

**FIGURE 16:**
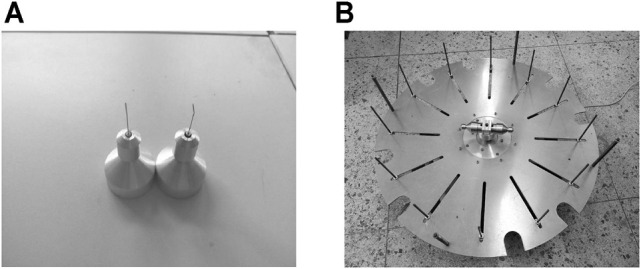
Nozzles and device of force-spinning. **(A)** Nozzles of force-spinning. **(B)** Device of force-spinning.

The comparative spinning experiments were carried out to test the correctness of the optimization. The polyethylene oxide (PEO) has a variety of outstanding properties, such as low toxicity and complete water solubility, excellent solution rheology, combination with organic solvents, low ash content, and thermo-plasticity, and can be used as a water-soluble film, textile slurry, thickener, flocculant, lubricant, dispersant, water-phase drag-reducing agent, cosmetic additive, and antistatic agent. The previous studies have shown that fibers can be spun more easily using the concentration of PEO solution between 4 wt% and 6 wt%; thus, the nanofibers were prepared with 5 wt% polyethylene oxide (PEO) aqueous solution (the molecular weight of PEO is 2 × 10^6^) under the motor speed of 3000 rpm using two kinds of nozzles. The spinneret used in this experiment cannot continuously provide the spinning solution, and the solution needs to be readded when the whole solution in the container is finished. A device capable of providing the continuous spinning solution is patent-pending (202110649108.3). The outlet diameter of unoptimized nozzles is 0.6 mm, while the outlet diameter of the optimized nozzle is 0.6 mm, the bending angle is 9.1°, and the curvature radius is 10 mm. The nozzles and the spinnerets of force-spinning are shown in Figure 16. The spinneret relates to fitting fixed above the motor shaft with four screws. The spinneret rotates with the electric motor at high speed. The operating speed of the motor is dominated by the current output, which is controlled by adjusting the high-frequency regulator. The motor speed increases 300 rpm each time when it starts to work, while the speed increases 10 rpm each time when the motor speed is less than 500 rpm than and spinning speed. The collected fibers are sampled and examined under a scanning electron microscope (SEM).

**FIGURE 17:**
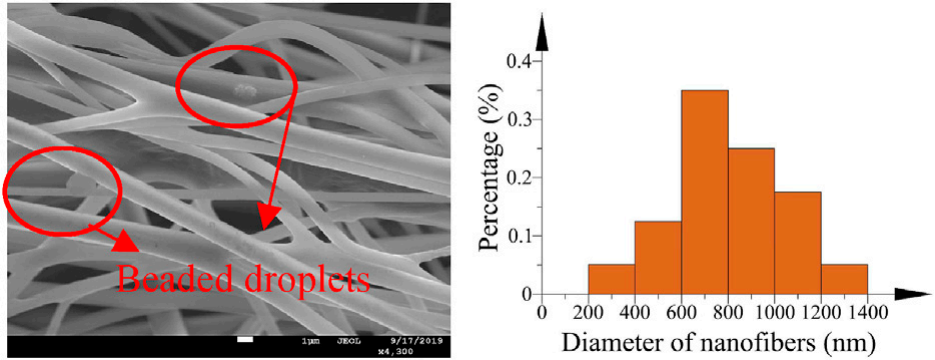
SEM image and the diameter distribution of nanofibers (unoptimized).

The morphology and diameter distribution of the nanofibers were visualized by SEM. Figure 17 shows the nanofibers fabricated in the straight tube. It is obvious that the fabricated nanofibers express worse morphology. Beaded droplets appear on the fibers, which makes the surface quality of fibers decrease sharply. It can be found that the diameter distribution of fibers is dispersed. The fiber diameter is mainly distributed between 400 and 1,200 nm.

**FIGURE 18:**
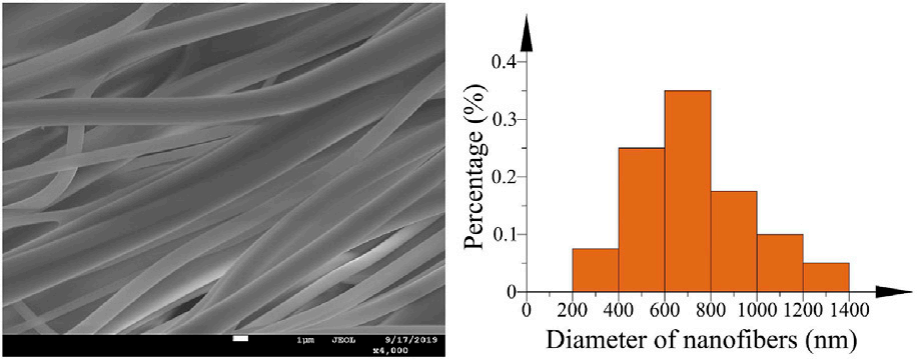
SEM image and the diameter distribution of nanofibers (optimized).

The fibers fabricated by optimized bent tube are shown in Figure 18 and they possess smoother surface and more even diameter that the diameter distribution of nanofibers is relatively in the range of 400–800 nm. Experiment results show that the morphology and the surface quality of nanofibers in the optimized bent-tube nozzles improve dramatically and the fibers with a smaller diameter can be obtained.

## Conclusion

This article analyzed the reasons for the uneven distribution of the solution outlet velocity in the outlet cross section and established the corresponding solution flow velocity model for each part by analyzing the spinning solution force in the four areas of the container, nozzle, straight tube, and bent tube. The factors mainly affecting the solution outlet velocity were determined, and the GWA was used to seek the optimal parameters. The morphology and diameter distribution of the fibers spun by optimized and unoptimized nozzles were compared. The results showed that the nozzle with an outlet diameter of 0.6 mm, a bending angle of 9.1°, and a curvature radius of 10 mm could fabricate fibers with better surface quality and smaller diameter distribution. However, the radial offset produced by the Coriolis force cannot be found. The influences of friction resistance and gravity on spinning solution flow were not considered in the theoretical derivation. In the next study, the influence of the above factors will be considered and applied to optimize the nozzle parameters for fabricating core/shell configuration fibers.

## Data Availability

The original contributions presented in the study are included in the article/Supplementary Material; further inquiries can be directed to the corresponding author.
